# Inhibition of Enveloped Viruses Infectivity by Curcumin

**DOI:** 10.1371/journal.pone.0062482

**Published:** 2013-05-01

**Authors:** Tzu-Yen Chen, Da-Yuan Chen, Hsiao-Wei Wen, Jun-Lin Ou, Shyan-Song Chiou, Jo-Mei Chen, Min-Liang Wong, Wei-Li Hsu

**Affiliations:** 1 Department of Veterinary Medicine, National Chung Hsing University, Taichung, Taiwan; 2 Graduate Institute of Microbiology and Public Health, National Chung Hsing University, Taichung, Taiwan; 3 Department of Food Science and Biotechnology, National Chung Hsing University, Taichung, Taiwan; Queensland Institute of Medical Research, Australia

## Abstract

Curcumin, a natural compound and ingredient in curry, has antiinflammatory, antioxidant, and anticarcinogenic properties. Previously, we reported that curcumin abrogated influenza virus infectivity by inhibiting hemagglutination (HA) activity. This study demonstrates a novel mechanism by which curcumin inhibits the infectivity of enveloped viruses. In all analyzed enveloped viruses, including the influenza virus, curcumin inhibited plaque formation. In contrast, the nonenveloped enterovirus 71 remained unaffected by curcumin treatment. We evaluated the effects of curcumin on the membrane structure using fluorescent dye (sulforhodamine B; SRB)-containing liposomes that mimic the viral envelope. Curcumin treatment induced the leakage of SRB from these liposomes and the addition of the influenza virus reduced the leakage, indicating that curcumin disrupts the integrity of the membranes of viral envelopes and of liposomes. When testing liposomes of various diameters, we detected higher levels of SRB leakage from the smaller-sized liposomes than from the larger liposomes. Interestingly, the curcumin concentration required to reduce plaque formation was lower for the influenza virus (approximately 100 nm in diameter) than for the pseudorabies virus (approximately 180 nm) and the vaccinia virus (roughly 335 × 200 × 200 nm). These data provide insights on the molecular antiviral mechanisms of curcumin and its potential use as an antiviral agent for enveloped viruses.

## Introduction

Curcumin (diferuloylmethane), a natural compound derived from turmeric (*Curcuma Longa*) is a widely used spice and coloring agent in food [1]. Accumulating evidence suggests that curcumin displays a number of pharmacological activities, including antiinflammatory [2], antioxidant [3] and antitumor [4], [5] activities. Recent studies have also shown that curcumin has antiviral activity [6], [7], [8], [9], [10]. In the study by Mazumder et al., curcumin inhibited HIV replication [11]. The specific interaction of curcumin with the viral proteins integrase and protease, which play central roles in viral replication, might represent the underlying mechanism for this effect [12]. Kutluay et al. also reported that curcumin treatment inhibited herpes simplex virus (HSV) immediate-early gene expression, possibly by interfering with the recruitment of RNA polymerase II to immediate-early gene promoters [13]. In other previous studies, curcumin inhibited several intracellular signaling pathways, including the Mitogen-activated protein kinase (MAPKs), phosphoinositide 3-kinase/protein kinase B (PI3K/PKB), and nuclear factor kappa B (NF-κB) pathways [14], [15], [16], and dysregulated the ubiquitin proteasome system (UPS) [17]. Activation of the NF-κB pathway is involved in the efficient replication of hepatitis C (HCV) [18] and influenza [19] viruses. Mazur et al., reported that treatment of influenza infection with NF-κB inhibitors downregulated influenza virus replication significantly [20]. However, Kim et al. described that curcumin inhibits HCV replication by suppressing the activation of Akt-SREBP-1, not through the NF-κB pathway [21]. In a recent study, curcumin decreased coxsackievirus B3 (CVB3) infection by dysregulating the UPS, a system required for CVB3 replication [6]. Overall, the finding from other research groups suggested that curcumin exerts antiviral activity through different mechanisms in different viruses; these mechanisms involve a direct inhibition of viral replication machinery or suppression of a cellular signaling pathway essential for viral replication.

In our previous study, treatment of cells with curcumin prior to infection markedly reduced the influenza A virus (IAV) yield at subcytotoxic doses [10]. This suggested that one of curcumin’s effects are mediated through the suppression of cellular signaling, possibly the NF-κB pathway. More strikingly, adding curcumin to the cell medium during viral adsorption inhibited virus production, and influenza virus exposed to curcumin before infecting MDCK cells markedly inhibited plaque formation [10]. By means of hemagglutination inhibition (HI) assays further demonstrated that curcumin interferes with HA receptor binding activity. Collectively, these assays implicated that curcumin might directly or indirectly interact with viral particles to interrupt early stage of IAV infection.

It was shown that curcumin influences a wide range of membrane proteins, by modulating the properties of the host lipid bilayer [22], [23]. As the HA protein is expressed on the envelope of the influenza virus, it is worthwhile to explore whether curcumin’s inhibitory effect is only on the HA activity of the influenza virus or it is deleterious to membrane proteins of other viruses. In this work, the effects of curcumin on the infectivity of several viruses were investigated and their potential underlying mechanisms were also discussed.

## Materials and Methods

### Cell Culture and Virus Infection

Madin-Darby canine kidney (MDCK; CCL-34) cells and African green monkey kidney cells BSC-1 (CCL-26), bought from American Type Culture Collection (ATCC), and Vero cells (ATCC CCL-81), a gift from Dr. S.S. Chiou, Graduate Institute of Microbiology and Public Health, Natinoal Chung-Hsing University (NCHU), were cultured in minimal-essential medium (MEM) with 10% heat-inactivated fetal bovine serum (FBS), penicillin 100 U/ml, and streptomycin 10 µg/ml. Porcine kidney (PK-15; BCRC 60057) cells, originally obtained from Bioresource Collection and Research Center (BCRC) of the Food Industry Research and Development Institute of Taiwan, were maintained in Dulbecco’s Modified Eagle’s Medium (DMEM, Hyclone) supplemented with penicillin 100 U/ml, streptomycin 10 µg/ml, and 5% of heat-inactivated FBS. Before infection, cells were washed with PBS and cultured in medium supplemented with antibiotics but without FBS (addition of 1 µg/ml of Worthington trypsin for influenza virus).

Human influenza virus PR8, A/Puerto Rico/8/34 (H1N1), was amplified in MDCK cells. Viruses for haemagglutination inhibition (HI) test were amplified in 10-day-old embryonated chicken eggs at 37°C for 2 days. The allantoic fluid was harvested and the titer of viruses was determined by standard plaque formation assay or hemagglutination (HA) test. Vaccinia virus, kindly provided by Dr. L Tiley in Department of Veterinary Medicine, Cambridge, UK, was grown in BSC-1 cells. Enterovirus 71 viruses (EV71), a gift from Dr. C. W. Lin, Department of Medical Laboratory Science and Biotechnology, China Medical University, were amplified in Vero cells. Pseudorabies viruses (PRV), obtained from Dr. T.J. Chang, were cultured in PK-15 cells. Japanese encephalitis virus (JEV) and Dengue virus type II (DVII), provided by Dr. S. S. Chiou, Graduate Institute of Microbiology and Public Health, NCHU, were cultured in Vero cells. Newcastle Disease viruses (NDV) for HA inhibition test were kindly provided by Dr. J. H. Shien, in Department of Veterinary Medicine, NCHU.

### Chemicals

Curcumin, obtained from Sigma-Aldrich, were dissolved in dimethyl sulfoxide (DMSO) at a stock concentration of 100 mM and stored in −80°C and were freshly diluted with infection medium prior to experiment.

### Cytotoxicity Test

Curcumin, 100 mM dissolved in dimethyl sulfoxide (DMSO), were freshly diluted with medium prior to experiment. 1.5×10^5^ of cells grown in 24-well plates for 24 hours were washed with PBS and were treated with serially diluted curcumin or DMSO at 37°C, 5% CO_2_ for 24 hours. Amplification of cells was measured directly by the total cell counts and the survival rate was estimated by the ratio of living cells/total cell counts by staining of 0.4% trypan blue. The cytotoxicity was estimated by comparison of the cell survival rate of curcumin-treated cells with that of DMSO-treated. The mock- treatment control was arbitrary set as 100%.

### Plaque Assay

MDCK cells grown to 80% confluence were washed twice with PBS followed by infection with serial dilutions of supernatants containing virus progenies in infectious medium i.e. MEM supplemented with 1 µg/ml of trypsin (Worthington, Freehold, NJ, USA) and antibiotics. After 2 hours absorption at 37°C, unbound viruses inoculums were removed and cells were then cultured with 1 ml/well MEM supplemented with 0.6% agarose at 37°C, 5% CO_2_ for 2 days. Viral plaques were visualized by staining with Giemsa (Sigma).

### Time-of-drug Addition Test on JEV and Dengue Virus Infection

Ten microliter of Curcumin (or DMSO, the solvent control) or were added to the medium at various times of JEV infection (200 pfu). Briefly, (1) full-time treatment: curcumin was included in the cell culture medium for 8 h and throughout the time of infection; (2) co-treatment: curcumin mixed with JEV in the infection medium was simultaneously added onto the cells and left on the cells throughout; (3) post-infection: curcumin was added to cells at 2 hours post infection and remained throughout the time of infection. After virus adhesion, virus inoculums were removed and vero cells were cultured in MEM (without FBS) containing 1% methylcellulose. The infectivity was measure according to the plaque formation units.

### Plaque Reduction Assay

To estimate the infectivity of viruses after curcumin treatment, 2000 pfu of PR8, vaccinia virus, EV71, JEV, DVII, or PRV particles were pre-incubated with 30 µM (unless otherwise stated) of curcumin at room temperature for one hour. Remaining infectivity after curcumin treatment was determined by plaque assay. To be able to measure the plaque formation number, the curcumin-treated viruses mix was further diluted to 10^−1^, 10^−2^, 10^−3^ with medium without FBS and added to appropriate cell lines. After 1 hour absorption at 37°C, the virus inoculums were removed and cells were then cultured with 1 ml/well MEM supplemented with 0.6% agarose for influenza (or medium containing 1% methylcellulose for other viruses) at 37°C, 5% CO_2_ for 2 days or until the plaque was visible. Viral plaques were fixed by methanol and stained with crystal violet (Sigma).

### Hemagglutination Inhibition (HI) Test

The hemagglutination (HA) titer of virus stock were initially determined by standard HA assay and 4 HA units (HAU) of viruses were then used for HI test. Briefly, curcumin stocks were diluted in PBS to a concentration of 1 mM (the highest concentration in the assay), and serial dilutions of curcumin were prepared by addition of 25 µl curcumin (1 mM) into the well of round-bottomed 96-well micro-plates, followed by 2-fold serial dilutions with PBS for 7 times. Twenty-five µl of virus stock were added into each well and incubated at room temperature for 1 hour. Subsequently, 50 µl of 0.75% chicken erythrocyte stocks were added to each well. The hemagglutination reaction was observed after 30 min incubation.

### Transfection Test

Cells were seeded into 24-well plates and grown to 90% confluence. The 4 µl of Cellfectin (Invitrogen, Carlsbad, CA, USA) and 0.8 µg of pEGFP-C1 plasmid (Clontech) were each diluted with 100 µl of Opti-MEM (Invitrogen, Carlsbad, CA, USA). Both of the diluted components were mixed and incubated at room temperature for 20 min. Subsequently, 30 µM or DMSO was added to the transfection reagent/DNA mixture and kept at room temperature for 40 min before added to the cell monolayer. The transfection efficiency and the intensity of GFP in each cell were recorded by flow cytometry analysis and fluorescent microscopy at 24-hour post transfection.

### Flow Cytometry Analysis

Transfected cells were washed with PBS and harvested in 1 mL of PBS. The signal of eGFP was then analyzed at least 10000 cells using FL-1 filter by FACS Calibur flow cytometer (BD Biosciences, MA, USA).

### Preparation of Liposome

The fluorescent SRB dye-loaded liposomes were prepared by a hydration/freezing and thawing/extrusion method [24]. The mixture of DPPC, DPPG, and cholesterol in a molar ratio of 45∶5:50 was dissolved in a solution of 6 ml chloroform, 1 ml methanol, and dried in a rotary evaporator. The dried lipid film was hydrated by the addition of 3 ml of 0.15 M SRB solution (in 0.02 M HEPES, pH 7.5, osmolality 530 mmol/kg). The lipid solution was processed with 5 freeze and thaw cycles, and then dependent on the final size of the liposomes desired, the liposomes were sequentially extruded by passing through a mini high-pressure extruder (Avanti Polar Lipids, Inc., AL, USA) with different pore-sizes (400, 200, and then 100 µm) of polycarbonate membrane (Whatman, NJ, USA). Unencapsulated SRB was removed by gel filtration using a Sephadex G-50 column with Tris-buffered saline (TBS: 0.02 M Tris with 0.15 M NaCl, 0.01% NaN3, pH 7.5) containing sucrose (osmolality 530 mmol/kg). Finally, the size of the liposomes was confirmed by a dynamic light-scattering measurement using a nanosizer 90ZS (Malvern Instruments, Worcestershire, UK).

### Treatment of Liposome with Curcumin

Liposome diluted in TBS (50 mM Tris base and 150 mM NaCl, osmolality 530 mmol/kg) was incubated with various concentrations of curcumin, DMSO (negative control), or 15 mM n-octylglucoside (n-OG), a detergent serving as positive control, at room temperature for one hour. The leakage of Sulforhodamine B (SRB) fluorescence was detected by SpectraMax M2e Microplate Reader (Molecular Devices, Inc., California, United States) at excitation wavelength of 490 nm.

To test whether influenza virus can reverse the curcumin’s effect on liposome, we added 2 different doses of influenza virus particles (2000 PFU and 10 000 PFU) to curcumin and SRB-loaded liposomes, and then detected fluorescence after 1 h incubation.

### Time Course Assay of Curcumin Pre-treatment

PR8 virus particles (2000 pfu) were mixed with 30 µM of curcumin in 200 µl. At 0, 5, 10, 20, 40, 60 min after curcumin treatment, an aliquot of 5 µl was added to MDCK cells in a well containing 495 µl infectious medium to make the curcumin concentration below viral inhibitory effect (i.e. 0.3 µM). The infectivity of PR8 was determined by the standard plaque assay to determine the infectivity of influenza virus.

### Characterization of Curcumin Effect on Plaque Formation Ability

PR8 virus particles (2000 pfu) were pre-incubated with 30 µM (unless otherwise stated) of curcumin at room temperature for one hour. Subsequently, the curcumin-virus mixture was diluted with fresh infectious medium to the concentration of 6 µM, 3 µM, 1.5 µM and kept at room temperature for one hour followed by the standard plaque assay.

### Replication of Curcumin-treated Viruses in Embryonated Eggs

The experiment protocol was approved by the Committee on the Ethics of Animal Experiments of National Chung Hsing University (Approval No: 97-91). The Embryonated hen’s eggs were purchased from the Livestock Research Institute, Chunan, Taiwan and incubated in hatchery until 10-day-old. Fifty or 5000 pfu of PR8 viruses were incubated with 30 µM of curcumin in a total volume of 500 µl infectious medium. One hour after incubation, inoculums were injected into the allantoic sac of embryonated hen’s eggs. Treated eggs were incubated in 37°C incubator for 18 or 24 hours, the yield of virus progeny was determined by HA test.

### Statistical Analysis

All data were calculated by Microsoft Excel. Results from at least three independent experiments were reported as mean values ± mean of standard deviations (S.E.M.).

## Results

### Curcumin blocked HA Activity of Newcastle Disease Virus (NDV)

**Figure 1:**
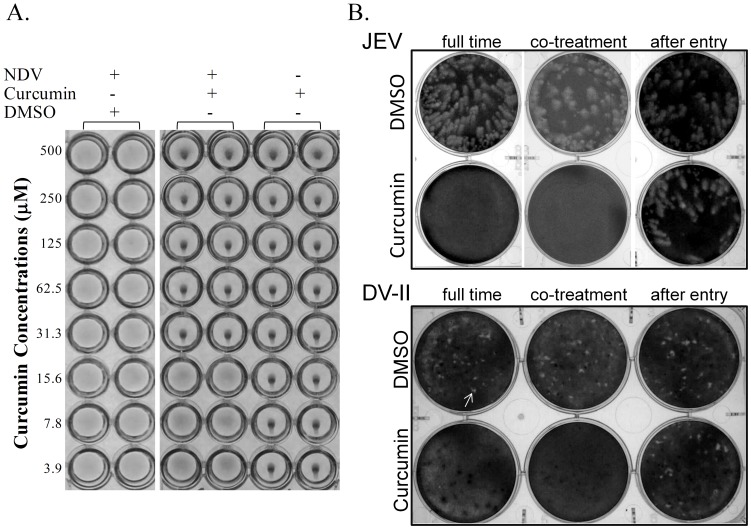
Treatment of curcumin reduces infectivity of enveloped viruses. (A) 4 HA units of Newcastle disease viruses (NDV) were incubated with 2-fold serially diluted curcumin or DMSO (vehicle control) and the hemagglutination inhibitory activity of curcumin was tested by incubation with chicken RBC at room temperature for 30 minutes. (B) time-of-drug addition test: 10 µM of curcumin or DMSO (as solvent control) was included into culture medium at various time points of Japanese encephalitis virus (JEV) or Dengue virus (DV-II) infection (200 pfu), for instance: (1) full time treatment: curcumin was added to vero cells at 8-hour prior to infection and included throughout the time of infection, (2) co-treatment: curcumin mixed with virus in the infection medium was added simultaneously to the cells and left on the cells throughout; (3) after-entry: curcumin was added to cells at 2 hpi and remained throughout the time of infection. Small-sized plaque of DV-II was indicated by arrowhead. Consistent results were observed from at least three independent experiments.

In our previous study, curcumin treatment abrogated the HA activity of the IAV subtypes H1N1 and H6N1 [10]. In this study, we treated paramyxovirus NDV, another virus that displays HA activity, with curcumin to determine if its effect is specific to the influenza virus. We incubated 4 HA units of NDV with various concentrations of curcumin for 60 min at room temperature and then assessed red blood cell (RBC) agglutination. Results showed that curcumin pretreatment (at concentrations of 31.2 µM or higher) inhibited the binding of NDV to chicken RBCs, as indicated by the spot-like appearance of non-hemagglutinated cells (Fig. 1A).

### Curcumin Inhibits Plaque Formation in Enveloped Viruses

It was indicated that curcumin modifies the lipid bilayer and influences membrane protein function [22]. The viral envelope is membranous structure; therefore, a time-of-drug addition test was employed to determine whether curcumin can also inhibit other enveloped viruses. Accordingly to the cytotoxicity test, 30 µM of curcumin slightly inhibit the growth of vero cells (Fig. S1), and therefore 10 µM curcumin was used for this assay. As indicated in Fig. 1B, including of curcumin throughout the time of infection (i.e. full-time treatment) completely abrogated the infectivity of two flaviviruses, JEV and Dengue (type 2; DV-II). Noticeably, addition of curcumin upon the viral attachment (i.e. co-treatment) pronouncedly inhibited both JEV and DV-II plaque formation; to a similar extent of full-time treatment. In contrast, no effect was observed, when curcumin was added after viral entry. These findings are consistent with the effect on influenza virus that curcumin blocks the virus infectivity by direct or indirect interfering the function of envelope protein. Since HA activity of NDV was also inhibited by curcumin, we then wonder whether curcumin generally affects the infectivity of enveloped viruses.

**Figure 2:**
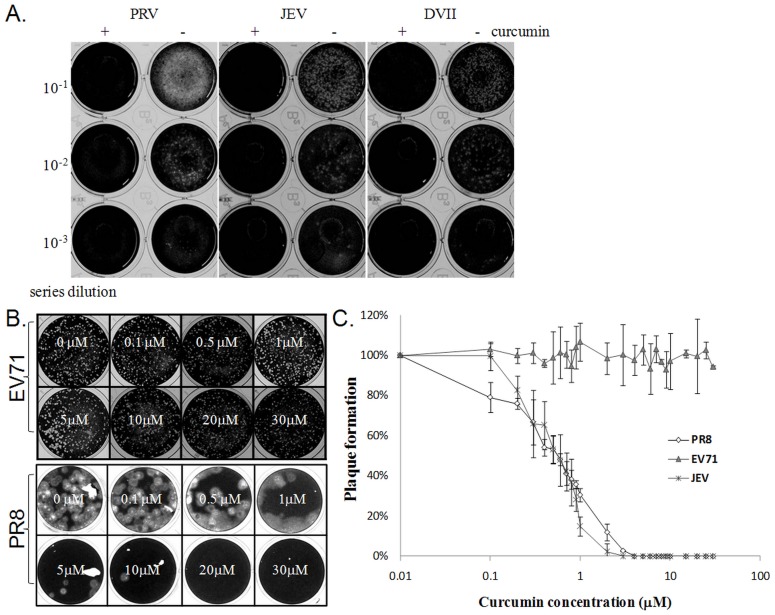
Pre-treatment of curcumin strongly inhibited enveloped viruses, but does not affect plaque formation of enterovirus 71 (EV 71). (A) 2,000 pfu of Pseudorabies virus (PRV), Japanese encephalitis virus (JEV), and Dengue virus serotype II (DVII) were pre-treated with 30 µM of curcumin for one hour and remaining viral infectivity was measured by standard plaque assay. To count plaque numbers, after one hour incubation, the mixture of virus and drug was further diluted into 10^−1^, 10^−2^, 10^−3^ with medium without serum followed by standard plaque assay. White spots indicate viral plaques. (B–C) To measure the effect of curcumin, 2,000 pfu of EV71, JEV, and influenza virus, strain PR8 were pre-treated with a serial dilutions of curcumin (30, 20, 10, 5, 1, 0.5, 0.1 µM to 0 µM) for one hour and the plaque formation ability was measured by standard plaque assay. Plaque formation ability of EV71 as not inhibited by curcumin, whereas infectivity of influenza viruses was strongly affected (B). Pre-treatment of curcumin inhibit plaque formation of JEV and Influenza to a similar extent, whereas EV71 remained unaffected (C). The results from Fig. 2C were plotted based on three independent experiments.

Since IAV, NDV and flavivirus analyzed in this assay were all RNA viruses, one enveloped DNA virus, pseudorabies virus (PRV swine herpes virus) was then used to test the possibility. The effect of curcumin on the infectivity of enveloped viruses was evaluated by the plaque formation assay. As shown in Fig. 2A, as with that of JEV, and DV-II viruses, pretreatment of PRV, with 30 µM of curcumin strongly inhibited plaque formation.

To determine whether the effects on the infectivity is specific to viruses with envelop, a nonenveloped virus, enterovirus 71 (EV71) was then included in the same set of test. For comparative analysis, 2000 PFU of EV71, influenza virus and JEV were incubated with different concentrations of curcumin and viral viability was evaluated using plaque assay. In contrast to the observations in the IAV and other enveloped viruses (PRV, JEV, and DV-II), EV71 plaque formation remained unaffected by curcumin at all analyzed concentrations (Fig. 2B) and similar inhibition effect was observed in IAV and JEV (Fig. 2C).

### Curcumin Disrupts the Integrity of Liposomes

**Figure 3:**
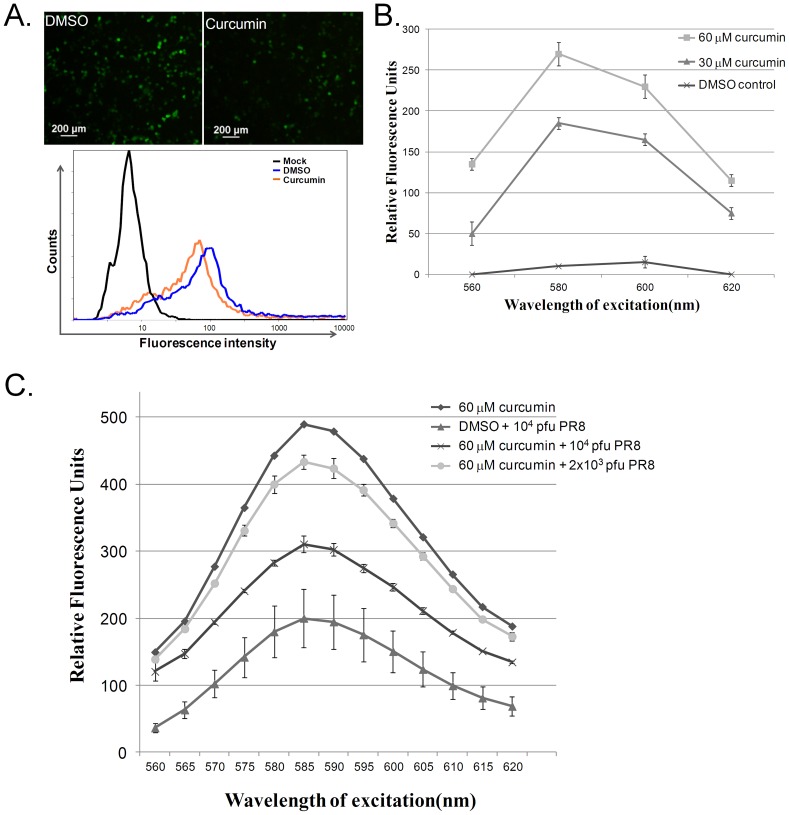
Curcumin affects the DNA transfection and structure of liposomes. The effect of curcumin on membrane structure was tested in two systems, commercial liposome-based transfection reagent (A) and Sulforhodamine B (SRB)-loaded liposome (B and C). (A) Curcumin was incubated with the mixture of eGFP plasmid (Clontech) and Cellfectin (Invitrogen) at room temperature for 40 min before added to the cell monolayer. At 24-hour post transfection, the transfection efficiency and the intensity of GFP in individual cells were recorded by fluorescent microscopy (upper panel) and flow cytometry analysis (lower panel). The images were taken under the same setting. (B) SRB-Liposomes were incubated with various concentrations of curcumin (30 µM, 60 µM), or DMSO (the solvent control) at room temperature for one hour followed by detection of SRB fluorescence (C) SRB-Liposome was incubated with two different doses of PR8 influenza viruses (2,000 and 10,000 pfu) and curcumin (60 µM), or DMSO at room temperature for one hour. The leakage of SRB fluorescence was detected by SpectraMax M2e Microplate Reader (Molecular Devices, Inc., California, United States) at excitation wavelength of 490 nm. The results from Fig. 3B and 3C were plotted based on three independent experiments.

Because curcumin had significant effects on HA protein function in 3 enveloped viruses (2 IAV subtypes and NDV) and also on the viability of 4 enveloped viruses, we further evaluated the effects of curcumin pretreatment on the integrity of the viral envelope using liposomes, a simple lipid structure that mimics the viral envelope. This was performed using a commercially available liposome-based transfection reagent and a sulforhodamine B (SRB)-loaded liposome. In transfection-based system, the transfection efficiency and reporter gene (green fluorescence protein; GFP) expression level in cells would indicate the function of liposome that reflects the integrity of liposome structure under curcumin treatment. As shown in Fig. 3A, incubation of the liposome/DNA mixture with curcumin (30 µM) markedly decreased the overall transfection efficiency and reduced the GFP signal in transfected cells compared with the DMSO solvent-control cells. Consistent results were observed in fluorescent SRB-loaded liposomes. We observed minimal fluorescence when the SRB was encapsulated in the liposomes. However, following membrane disruption and the subsequent release of SRB from the liposomes, fluorescence emission (590 nm wavelength) was detectable. Liposomes treated with 30 µM curcumin displayed higher levels of SRB fluorescence than DMSO-treated liposomes, indicating that curcumin induces leakage of the fluorescent dye (Fig. 3B). We observed a higher level of SRB leakage from the liposomes treated with 60 µM curcumin than from those treated with 30 µm curcumin.

Liposome, as a simpler membrane structure, was used to mimic envelop structure (Fig. 3A, and B). We then further conducted influenza virus competition test to evaluate if the SRB-loaded liposomes is an adequate model to represent the viral envelope during curcumin treatment. Presumably if liposome and viral envelop are similar in terms of lipid structure, then addition of virus particles was supposed to compete the effect of curcumin on liposomes. We added 2 doses of influenza virus particles (2000 PFU and 10 000 PFU) to curcumin and SRB-loaded liposomes, and then detected fluorescence after 1 h incubation. The liposomes treated with curcumin alone displayed highest SRB fluorescence. Addition of influenza virus reduced the curcumin-induced SRB leakage (Fig. 3C), with higher doses of IAV more potently reducing the effects of curcumin on SRB leakage.

### Curcumin’s Effects on SRB Leakage are Size-dependent

**Figure 4:**
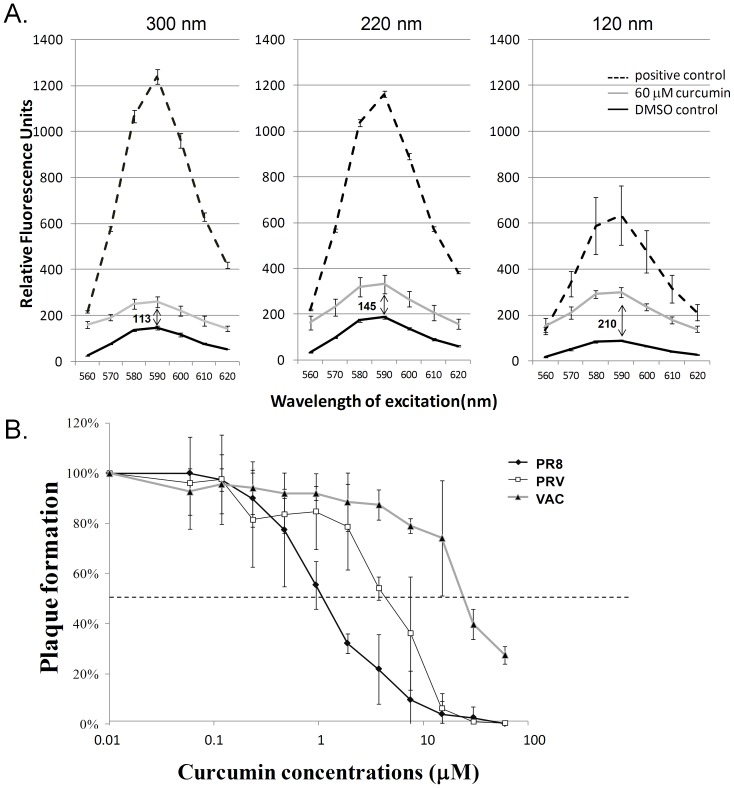
Effect of curcumin on liposomes and viruses with different sizes. (A) Three different diameters of SRB-Liposome i.e. 300 nm, 220 nm, 120 nm were incubated with curcumin (60 µM), DMSO (the solvent control), or 15 mM n-octylglucoside (n-OG), a detergent serving as positive control, at room temperature for one hour. The leakage of SRB fluorescence was detected by SpectraMax M2e Microplate Reader (Molecular Devices, Inc., California, United States) at excitation wavelength of 490 nm. (B) Effects of curcumin on plaque formation of enveloped viruses with different sizes. 2,000 pfu of influenza virus (strain PR8) and two DNA viruses, i.e. pseudorabies virus (PRV) and vaccinia viruses (VAC) were pre-treated with 30 µM of curcumin for one hour. The plaque formation ability was measured by standard plaque assay and plotted as a percentage of the untreated controls. Dash lines indicate reduction of plaque formation by 50% relative to the control group. Data are presented as mean values ± standard deviation (SD) from three independent experiments.

In previous plaque reduction assays, curcumin exerted significant antiviral effects at subcytotoxic concentrations, with a selective index of 92.5 [10]. Viral envelopes are derived from cell membranes; therefore, in this study, we investigated the mechanisms by which curcumin selectively disrupts lipid bilayers in different organelles. The diameter of the influenza virus (80–100 nm) is approximately 100-fold smaller than that of the mammalian cell (10 µm). We, thus, investigated the influence of the sizes of particles on curcumin’s effects. We treated similar number of SRB-containing liposome particles of 3 different diameters (300, 220, and 120 nm) with curcumin and evaluated the leakage of fluorescence. As shown in Fig. 4A, when normalized with the fluorescence unit in the DMSO control (by subtraction), we detected the lowest and highest levels of SRB leakage in liposomes 300 nm and 120 nm in diameter, respectively. This indicated that curcumin exerts more potent effects on liposomes with smaller diameters.

### Plaque Formation of Enveloped Viruses with Different Sizes is Influenced by Curcumin

**Figure 5:**
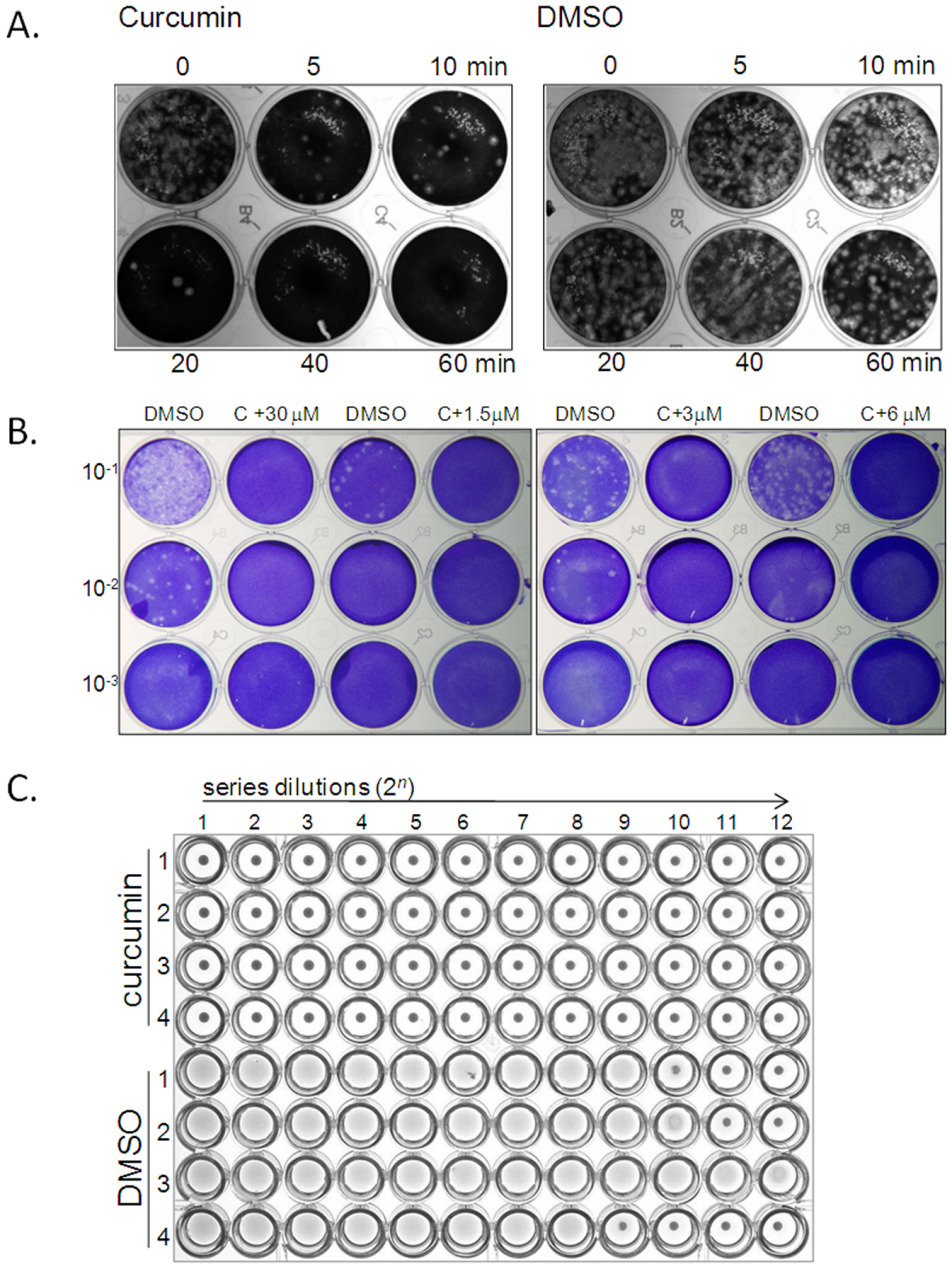
Effect of curcumin on inhibition of viral plaque formation and infectivity is irreversible. A time course treatment was conducted to determine the time required for plaque reduction (A). Influenza virus (50 pfu) was mixed with curcumin (30 µM) or DMSO (solvent control). At different periods of times, i.e. 0, 5, 10, 20, 40, 60 min of incubation, the virus-test drug mixtures were added to cells followed by standard plaque assay. To evaluate whether curcumin-induced inhibition of viral plaque formation can be restored, one hour after curcumin (30 µM) treatment, the influenza virus (2000 pfu of PR8) and curcumin mixture was subsequently diluted to final concentrations of curcumin at 6 µM, 3 µM, and 1.5 µM, followed by standard plaque assay (B). To test whether the curcumin-induced loss of virus infectivity is irreversible, 50 pfu of PR8 viruses were incubated with 30 µM of curcumin or DMSO in a total volume of 500 µl infectious medium. One hour after incubation, inoculums were injected into the allantoic sac of 4 embryonated hen’s eggs. Treated eggs were incubated in 37°C incubator for 18 hours, the yield of virus progeny was determined by HA test (C). Consistent results were observed from at least three independent experiments.

Envelope structure of viruses is much more complicated than liposome. The size dependent effect was then tested with viruses with various sizes. Our previous data has indicated that curcumin inhibited plaque formation in JEV and the influenza virus (similar size) to similar extents, with a minimal concentration for complete inhibition of 3 µM for JEV and 4 µM for influenza (Fig.2B). We further comparatively compared the effect of curcumin on other three enveloped viruses including the influenza virus (H1N1 subtype, PR8 strain), PRV, and vaccinia virus. The virions of influenza virus and PRV are spherical with diameters of approximately 80 nm to 120 nm, and 150 nm to 200 nm, respectively. Vaccinia virus, a large DNA virus, is brick-shaped and approximately 220 nm to 450 (length)×140 nm to 260 nm (width)×140 nm to 260 nm (height) in size [25]. Consistently, curcumin abrogated plaque formation in the influenza virus and PRV at concentrations ≥30 μµM. However, at concentrations ranging from 0.93 µM to 3.75 µM, curcumin treatment exerted more potent inhibitory effects on influenza virus infectivity than on PRV infectivity. The curcumin concentration required to reduce plaque formation by 50% relative to the control (EC_50_) was 1.15 µM for influenza and 4.61 µM for PRV (Fig. 5B). Interestingly, this size dependent effect was more apparent when comparing the inhibitory effects of curcumin on the influenza virus with those on the vaccinia virus. As shown in Fig. 4B, curcumin inhibited the infectivity of the vaccinia virus, an enveloped virus. However, none of the analyzed curcumin concentrations were able to fully abrogate vaccinia virus infectivity: the highest curcumin concentration (60 µM) reduced vaccinia viral plaque formation to 30% of that in the control experiment. This concentration was substantially higher than that required for 100% inhibition of JEV and influenza (Figs. 2B and 4B). This results supports our hypothesis that the requirement for a higher curcumin concentration to inhibit the infectivity of viral particles with larger diameters.

### Curcumin-induced Inhibition of Viral Plaque Formation is Irreversible

Curcumin might inhibit viral infectivity by disrupting membrane integrity or by interfering with membrane protein(s) function. This study evaluated the efficiency of curcumin’s viral inhibitory effects and also the reversibility of these effects. In plaque reduction assays, incubation of the influenza virus with curcumin (30 µM) for 1 h fully abrogated influenza virus infectivity (Fig. 2). We then conducted a time course treatment to determine the time required for plaque reduction. Results indicated that dramatic decrease of plaque formation was observed after virus was exposed to curcumin for 5 min; however a minimal curcumin treatment time of 40 min is required to completely abrogate plaque formation (Fig. 5A). To evaluate whether curcumin-induced loss of virus infectivity can be restored, after pretreatment of curcumin, we diluted the virus-curcumin mixture to subinhibitory doses of curcumin. As shown in Fig. 4B, the minimal concentration of curcumin required for complete inhibition of plaque formation of IAV was ∼4 µM. If this effect is reversible, after 1 h curcumin treatment, subsequent dilutions of the virus-curcumin mixture to concentrations less than 4 µM should restore infectivity to certain extents. Results showed that curcumin treatment, followed by serial dilutions to final concentrations of 6 µM, 3 µM, and 1.5 µM, did not reverse the effects of curcumin, as indicated by the lack of plaque formation (Fig. 5B). However, dilutions of the virus-solvent control (DMSO) mixture restored the marginal inhibitory effects of DMSO. We then used the embryonic chicken egg, a potent amplification vessel for the influenza virus, to investigate whether curcumin treatment irreversibility inhibited the influenza virus. Viruses pre-treated with curcumin (for one hour) were unable to amplify in embryonic eggs. However, eggs innoculated with a high dose (5000 PFU) of PR8 treated with DMSO produced 2^5.5^ HA units and 2^9.75^ HA units of viral progeny at 18 h and 24 h after infection, respectively (Table 1 and Fig. 5C). These results indicated that the inhibitory effects of curcumin on influenza virus infectivity are irreversible.

**Table 1:** Virus yields in embryonic eggs infected with influenza virus pre-treated with curcumin.

	Curcumin (+)		Curcumin (−)	
	Yield of virus progeny: HA titer (2^n^ *)		Yield of virus progeny: HA titer (2^n^ *)	
Virus used	18 hpi	24 hpi	18 hpi	24 hpi
**50 pfu**	0.00	0.00	0.00	8.25±1.71
**5000 pfu**	0.00	0.00	5.5±0.58	9.75±1.26

* The values of HA titer represent the mean ± S.E.M. for four independent experiments.

## Discussion

This study presents several novel findings. To our knowledge, it is the first to show that curcumin generally inhibits enveloped virus infectivity. In addition to inhibiting HA activity, a novel mechanism was investigated; as evidenced in the liposome-based assay systems, we proposed that the integrity of membrane structure, e.g., viral envelope, could be affected by curcumin treatment. As for the four enveloped viruses analysed in the current study, the EC_50_ of curcumin on inhibition of plaque formation for larger viruses is greater than that for smaller viruses.

Previous studies reported that curcumin associates with membranes [26], [27]. The hydrophobic properties of membranes favor the intercalation of curcumin into the lipid bilayer, such as in cellular membranes, where phenolic rings of curcumin are essential for interaction with hydrogen-bonding sites. Several studies also identified curcumin as a membrane-disturbing agent. Curcumin treatment induced alterations in membranous properties, including morphological changes, and increased permeability and fluidity. The interactions between the cell membranes and curcumin might have caused these effects [26], [28]. Jaruga et al. observed that treatment of erythrocytes with >100 µM curcumin concentrations induced changes in the integrity of their cell membranes [26], [27]. Similarly, at a high treatment concentration (5 mM), curcumin increased lactate dehydrogenase (LDH) leakage in rat hepatocytes (25%) compared with the LDH leakage observed in untreated cells (15%) [29]. Research using a rat thymocyte model further showed that curcumin is able to penetrate the cytoplasm and accumulate in membranous structures of intracellular organelles and the nuclear membrane. In addition to inducing morphological changes, curcumin treatment decreased mitochondrial membrane potentials [28]. In other studies, curcumin influenced the function of several proteins, such as epidermal growth factor receptor [30] and P-glycoprotein [31]. These results suggested that additional to curcumin’s effects on membrane structures, interactions between the phenolic groups of curcumin and hydrogen bonding sites in the cellular membranes could influence membrane activities or membrane protein function.

In our previous study, curcumin effectively interfered with the HA activity of the influenza virus [10]. In this study, HA activity was inhibited significantly in NDV treated with curcumin concentrations higher than 30 µM. The hemagglutinin-neuraminidase (HN) protein is responsible for the HA activity of NDV. Since the HN protein sequence and conformation are dissimilar from those of the HA protein of the influenza virus, inhibition of HA function in both viruses after curcumin treatment suggested that the HI effects might result from different mechanisms, or from a general disruptive effect on the viral envelope. Following the treatment of several enveloped viruses and one nonenveloped virus, EV71 (*Piconaviridae*) with curcumin, we observed that the effects of curcumin on inhibition of viral plaque formation are specific to enveloped viruses: the infectivity of EV71 remained unaffected by curcumin treatment. As the 7 enveloped viruses, including influenza A virus (H1 and H6 subtypes), pseudorabies virus, 2 flaviviruses (JEV and Dengue virus), vaccinia virus, and NDV, are classified into 5 families, it suggested that curcumin exerts a general inhibitory effect on viruses with an envelope structure. Given that pretreatment of the viruses with curcumin irreversibly abrogated plaque formation and HA activity, it indicated that curcumin could serve as a virucidal agent for enveloped viruses.

In this study, the concentration of curcumin required to inhibit viral HA activity (30 µM) is lower than that reported in our previous study [10], and those reported by other research groups as disruptive to erythrocyte membranes [26], [27]. In HI assays, we observed hemolysis at curcumin concentrations higher than 500 µM. Pretreatment of RBC with 30 µM curcumin had no effects on the HA activity of influenza viruses, indicating that the erythrocyte membrane remains intact at curcumin concentrations effective for HA inhibition. At concentrations lower than 30 µM, we observed insignificant cellular toxic effects. However, EC_50_ required for inhibition of influenza virus was approximately 0.47 µM (with a selective index, CC_50_/EC_50_, of 92.5) [10]. These observations indicated that, despite viral envelopes and cellular membranes being composed of a phospholipid bilayer, curcumin (30 µM) selectively inhibits the infectivity of virus particles by disrupting the function of viral enveloped proteins, such as the HA protein of the influenza virus and the HN protein of the NDV. Cell viability, however, remains unaffected by curcumin treatment. Several factors might contribute to curcumin’s disrupting effects on different viruses and cells, such as the complexities of surface protein compositions and the sizes of viral particles. Numerous surface proteins attach to cellular membranes, whereas few proteins anchor to the surface of viral particles: Three envelope proteins (HA, NA, and M2) on the influenza virus, two envelope proteins (HN and F) on NDV, and two proteins (E and M) on flaviviruses. Considering the essential roles of each of these viral proteins in viral infection, it is likely that interruption of the function of any viral surface protein by direct interaction with curcumin, or as a sequential effect resulting from viral envelope disturbance by curcumin, would have a more significant effect on virus infectivity than on the cell. In different viruses, 30 µM curcumin fully abrogated influenza A virus and JEV infectivity (Fig. 2B), but had minimal inhibitory effects on vaccinia virus infectivity (Fig. 4B). In general, compared with DNA viruses, RNA viruses have a smaller-sized genome, encoding fewer proteins and a simple envelope structure. Among the 4 viruses used for comparative analysis, both the influenza A virus and JEV are RNA viruses, with viral particles approximately 50 nm to 100 nm, and contain 2 or 3 envelope proteins. In contrast, the vaccinia viral particle is considerably much larger, 220 nm to 450 nm (length)×140 nm to 260 nm (width)×140 nm to 260 nm (height), with a more complex structure [32]. It is possible that interference of the envelope proteins has minimal detrimental effects on vaccinia virus replication, or that other proteins with similar functions can compensate for the loss of this activity. Similarly, this can explain curcumin’s selective inhibitory effects on viruses: the constitution of surface proteins on cell membrane is much more complicated than viral envelope, and the average diameter of a cell ranges from 10 µm to 50 µm; approximately at least 100-fold larger than that of influenza virus. At identical treatment concentrations, curcumin would, therefore, exert more potent effects on small virus particles than on cells.

Previous studies described the inhibitory effects of curcumin on several enveloped viruses (such as HIV, influenza A virus, herpes simplex virus, hepatitis B virus) [7], [8], [9], [10], [13] and on one nonenveloped virus, coxsackievirus B3 [6]. In this study, the infectivity of EV71, which is classified into the same genus as coxsackievirus B3 (*Picornaviridae* Enterovirus), remained unaffected by curcumin treatment. Different experimental designs could have caused these inconsistent findings. Assay systems (plaque reduction and HI) of this study evaluated the effects of curcumin on virus particles and did not consider its effects on cells. However, the study by Si et al. investigated the cellular effects of curcumin, observing the suppression of coxsackievirus B3 replication through dysregulation of the UPS [6]. These different observations indicate that curcumin inhibits viral infection through multiple mechanisms.

Accumulating evidence suggests the potential use of curcumin as an antiviral drug; however, the mechanisms underlying its broad spectrum biological effects have yet to be fully elucidated. Our findings indicate that curcumin has potential anti-viral activity for a variety of enveloped viruses analyzed in this study because of its membrane-disturbing (or membrane protein modification) properties. This novel finding implies that when evaluating the mechanisms of curcumin-induced antiviral activity based on the time-of-drug addition experiment, misinterpretation of the observations is possible. A typical experimental design for investigation of curcumin’s antiviral activity is the addition of curcumin to a cell culture medium prior to and/or during the course of infection [6], [13]. In the presence of curcumin, the effective viral load would decrease significantly during viral absorption (prior to viral entry). For example, simultaneous addition of the influenza virus and curcumin to a cell culture reduced the virus yield to <5% of that in the control, and viruses pre-exposed to curcumin prior to infecting MDCK cells markedly inhibited plaque formation. The initial reduction in the effective viral load would, thus, contribute to the reductions in virus yields. The investigation of selected cellular signaling proteins involved in curcumin-dependent antiviral activity could then be misleading because reductions in viral replication or yield might not be exclusively through cellular effects, also resulting from the effects of curcumin on virus particles during the early stage of infection. An appropriate experimental design for investigating the effects of curcumin on enveloped viruses should avoid simultaneous incubation of the test viruses with curcumin during viral absorption or pre-treatment of virus with curcumin. Curcumin could, however, be included in the cell culture: (I) before infection but removed upon viral absorption (i.e., treatment of the cells with curcumin) to evaluate the establishment of antiviral status in response to curcumin treatment; and (II) after fusion of the cell membranes with the viral envelope, or at selected time points after viral entry, to determine the effects of curcumin on viral replication procedures and to evaluate the contribution of cellular machinery during viral infection.

## Supporting Information

Figure S1 **Cytotoxicity test of curcumin.** Vero cells grown in 96-well (for MTT test) or 24-well (for cell survival analysis) plates for 16 hours were washed with PBS and were treated with DMSO (control) or curcumin at indicated concentrations at 37°C, 5% CO_2_ for 24 hours. Proliferation of cells was then measured by the standard MTT test (MTT obtained from Sigma-Aldrich) (A), or directly by the total cell counts (B). (A) For MTT test, cells were washed with PBS and were then treated with 100 microliter of MTT solution (0.5 mg/ml) for one hour. Subsequently, the blue crystals were solublized with 0.04 N HCl in absolute isopropanol and the intensity is measured colorimetrically at 570 nm. (B) Cell survival rate was estimated by the ratio of living cells/total cell counts after stained with 0.4% trypan blue. The cytotoxicity was estimated by comparison of the cell survival rate of curcumin-treated cells with that of mock-treated (0 µM). The mock- treatment control was arbitrary set as 100%. The results were plotted based on three independent experiments.
(TIF)
