# Supplementary figures and images for: Inhibition of Enveloped Viruses Infectivity by Curcumin
Source: PLoS One. 2013 May 1;8(5):e62482. doi: 10.1371/journal.pone.0062482 (PMC3641039; doi:10.1371/journal.pone.0062482)

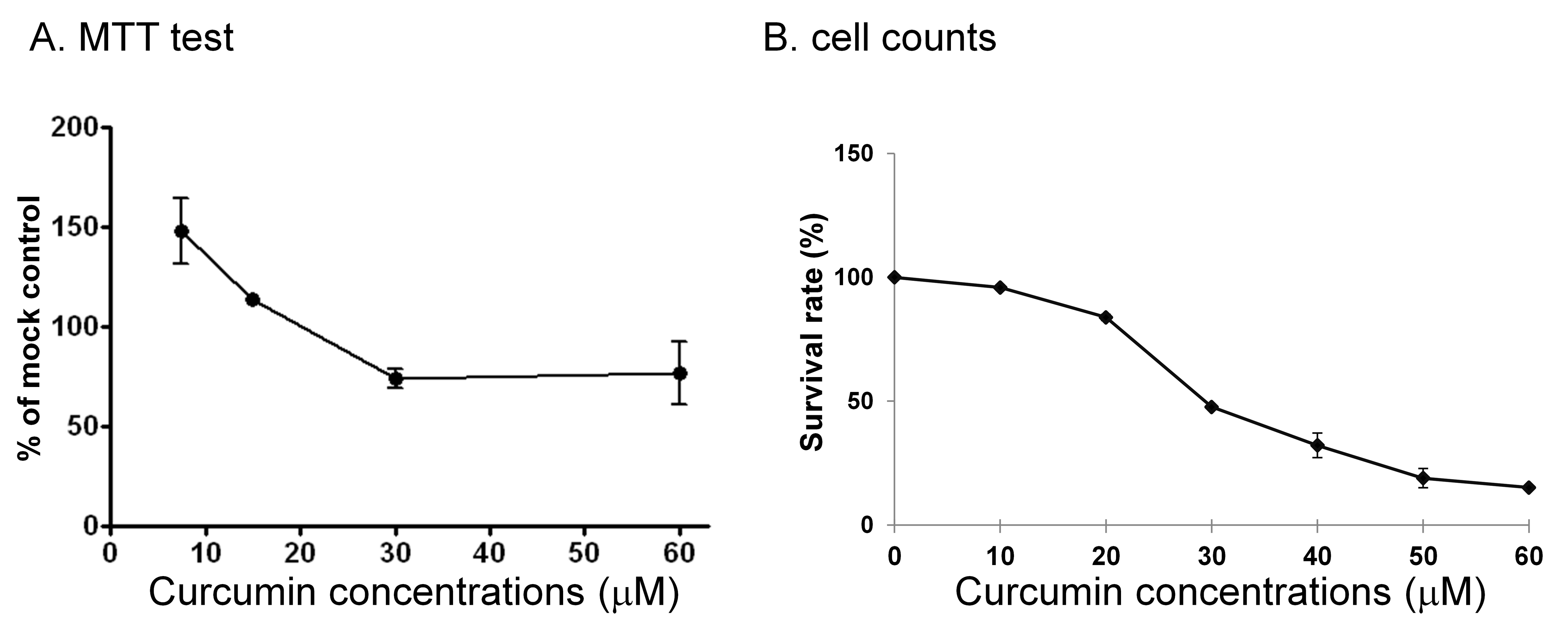

Supplement: Figure S1 — Cytotoxicity test of curcumin. Vero cells grown in 96-well (for MTT test) or 24-well (for cell survival analysis) plates for 16 hours were washed with PBS and were treated with DMSO (control) or curcumin at indicated concentrations at 37°C, 5% CO2 for 24 hours. Proliferation of cells was then measured by the standard MTT test (MTT obtained from Sigma-Aldrich) (A), or directly by the total cell counts (B). (A) For MTT test, cells were washed with PBS and were then treated with 100 microliter of MTT solution (0.5 mg/ml) for one hour. Subsequently, the blue crystals were solublized with 0.04 N HCl in absolute isopropanol and the intensity is measured colorimetrically at 570 nm. (B) Cell survival rate was estimated by the ratio of living cells/total cell counts after stained with 0.4% trypan blue. The cytotoxicity was estimated by comparison of the cell survival rate of curcumin-treated cells with that of mock-treated (0 µM). The mock- treatment control was arbitrary set as 100%. The results were plotted based on three independent experiments. (TIF) [file pone.0062482.s001.tif]
